# Comparative Evaluation of Dentinal Tubule Penetration, Void Formation, and Push-Out Bond Strength of Premixed Injectable Bioceramic Sealers and AH Plus

**DOI:** 10.7759/cureus.102804

**Published:** 2026-02-01

**Authors:** Bazila Malik, Divya Chowdhary, Sonali Taneja

**Affiliations:** 1 Department of Conservative Dentistry and Endodontics, I.T.S Centre for Dental Studies and Research, Ghaziabad, IND

**Keywords:** bioceramics, dentin, endodontics, epoxy resin-based root canal sealer, root canal sealer

## Abstract

Introduction: Successful root canal obturation requires a fluid-tight seal to prevent reinfection and promote healing. The aim of this study was to compare dentinal tubule penetration, void formation, and push-out bond strength of three premixed injectable bioceramic sealers (BioRoot Flow, ZenSeal, and Bio-C Sealer) with those of the conventional epoxy resin-based sealer AH Plus.

Materials and methods: Ninety-six extracted human mandibular premolars with straight single canals were decoronated to 14 mm root length and prepared using ProTaper Gold rotary files up to F3, followed by standardized irrigation. The teeth were randomly divided into four groups (n = 24 each): BioRoot Flow, ZenSeal, Bio-C Sealer, and AH Plus (control). All the canals were obturated using the single-cone technique with F3 gutta-percha cones. After two weeks of storage at 37 °C and 100% humidity, the specimens were sectioned. Dentinal tubule penetration (n = 8 per group) was evaluated using confocal laser scanning microscopy with rhodamine B-labelled sealers. The void area (n = 8 per group) was measured under a stereomicroscope using the ImageJ software. Push-out bond strength (n = 8 per group) was tested using a universal testing machine. The data were then analyzed (p < 0.05).

Results: BioRoot Flow showed significantly greater dentinal tubule penetration than the other sealers in the coronal, middle, and apical thirds (p < 0.001), followed by Bio-C, ZenSeal, and AH Plus (lowest). Void formation was significantly higher in AH Plus than in all bioceramic sealers (p < 0.001), with BioRoot Flow and Bio-C exhibiting the smallest number of voids. The push-out bond strength was the highest for AH Plus across all levels (p < 0.001), followed by BioRoot Flow, ZenSeal, and Bio-C. In all groups, penetration and bond strength decreased significantly from coronal to apical, while the void area showed the opposite trend.

Conclusion: Premixed injectable bioceramic sealers, especially BioRoot Flow, provided superior dentinal tubule penetration and significantly reduced void formation compared to AH Plus, with clinically acceptable bond strength. These findings support BioRoot Flow as an effective alternative for improving sealing performance in root canal obturation.

## Introduction

Successful root canal treatment relies on effective biomechanical preparation, thorough disinfection, and fluid-tight three-dimensional obturation to eliminate microorganisms, prevent reinfection, and promote periapical healing [1]. Gutta-percha remains the gold standard core material because of its biocompatibility and dimensional stability; however, it lacks chemical bonding to dentin, requiring a root canal sealer to seal irregularities, lateral canals, and dentinal tubules, while enhancing overall adaptation and microbial entombment [2].

For decades, epoxy resin-based sealers, such as AH Plus, have been widely regarded as the benchmark, offering low solubility, dimensional stability, and reliable long-term sealing. Despite these advantages, AH Plus presents limitations, including potential cytotoxicity, hydrophobic behavior that restricts penetration in moist environments, technique-sensitive mixing prone to voids, and challenges during retreatment [3].

These shortcomings have fueled the evolution of endodontic sealers toward bioceramic-based formulations, primarily calcium silicate-based materials, which exhibit bioactivity, biocompatibility, antibacterial properties, and moisture-activated setting. These sealers chemically bond to dentin via hydroxyapatite formation, creating a "mineral infiltration zone," which enhances interfacial sealing and tissue response [4]. Premixed injectable bioceramic sealers represent a significant advancement, providing consistent viscosity, syringe-based delivery, reduced procedural errors, and improved handling in complex anatomies compared to earlier powder-liquid or two-paste systems. However, both have comparable survival and success rates [5].

Among contemporary premixed injectable bioceramics, BioRoot Flow (Septodont), ZenSeal (Kerr), and Bio-C Sealer (Angelus) stand out for their hydrophilicity, fine particle size, and flowability, which promote deeper dentinal tubule penetration and superior adaptation. Key evaluation parameters include dentinal tubule penetration (increasing interfacial area and blocking microbial pathways), void formation (compromising seal integrity and permitting leakage), and push-out bond strength (reflecting resistance to dislodgement under functional loads) [6,7]. While AH Plus typically demonstrates superior adhesive strength due to its resin chemistry, bioceramic sealers often excel in tubule penetration and void reduction, particularly under moist conditions [8].

Although bioceramic sealers have gained clinical popularity with comparable treatment outcomes to resin-based sealers, limited comparative data exist on newer premixed formulations, such as BioRoot Flow and ZenSeal, relative to Bio-C and the established AH Plus benchmark. The aim of this study was to compare the sealing performance of three premixed injectable bioceramic sealers, BioRoot Flow, ZenSeal, and Bio-C, with that of the conventional resin-based sealer AH Plus. The specific objectives were to evaluate and compare the depth of dentinal tubule penetration using confocal laser scanning microscopy, assess and compare the area of void formation using stereomicroscopy and image analysis, and measure and compare the push-out bond strength to dentin using a universal testing machine.

## Materials and methods

Study design and setting

This in vitro comparative experimental study was conducted at the Advanced Research Laboratory of the Department of Conservative Dentistry and Endodontics, I.T.S Center for Dental Studies and Research, Muradnagar, Ghaziabad, Uttar Pradesh, India, from February 2024 to September 2024. The study protocol was reviewed and approved by the Institutional Ethical Committee of the Institute for Dental Studies and Research, Muradnagar, Ghaziabad (approval number: ITSCDSR/Director-Principal/2024/L/070 dated 31-01-2024). Written informed consent was obtained from all patients before the extraction of the teeth used for this study.

Sample size calculation

The required sample size was determined using the OpenEpi software (version 3.01; Emory University, Atlanta, GA, USA). Based on a one-way analysis of variance (ANOVA) F-test (fixed effects, omnibus) with an alpha error of 0.05 and a power of 80%, a minimum total sample size of 96 was determined. This was derived using a medium effect size (f = 0.35), which was obtained from a previous study evaluating the dentinal tubule permeability of bioceramic sealers [7]. The 96 samples were equally allocated to four experimental groups (n = 24 per group).

Specimen selection

Ninety-six freshly extracted human mandibular premolars with single, straight root canals, fully formed apices, and free from caries, cracks, restorations, resorption, or previous endodontic treatment were selected. Teeth showing curvature, calcification, or accessory canals on preoperative radiographs were excluded from the study. Immediately after extraction, soft tissue remnants were removed, and the teeth were stored in 0.1% thymol solution at room temperature to prevent microbial growth until further processing.

Specimen preparation

All selected teeth were decoronated at the cemento-enamel junction using a low-speed diamond disc under continuous water cooling to obtain standardized root lengths of 14 mm. The working length was established by advancing a size 10 K-file (Mani Inc., Tochigi, Japan) until its tip was visible at the apical foramen and then subtracting 1 mm. Root canal preparation was performed using ProTaper Gold rotary instrumentation (Dentsply Sirona, Ballaigues, Switzerland) up to the size of the master apical file F3. Irrigation between instruments was performed using 5.25% sodium hypochlorite (Septodont, Saint-Maur-des-Fossés, France). The final irrigation protocol included 17% ethylenediaminetetraacetic acid (EDTA) (Prevest DenPro, Jammu, India) for one minute, followed by 5.25% sodium hypochlorite (NaOCl) and a final rinse with saline. The canals were thoroughly dried using sterile paper points (Dentsply Sirona, Ballaigues, Switzerland).

Experimental groups

The prepared root specimens were randomly allocated to four experimental groups, with 24 teeth in each group (n = 24), according to the type of root canal sealer used for obturation. Group A consisted of specimens obtained using BioRoot Flow (Septodont, Saint-Maur-des-Fossés, France), which is a premixed injectable bioceramic sealer. Group B included specimens treated with ZenSeal (Kerr, Orange, CA, USA), a premixed bioceramic sealer. Group C comprised specimens obtained using Bio-C Sealer (Angelus, Londrina, PR, Brazil), whereas Group D, serving as the control group, utilized the conventional epoxy resin-based sealer AH Plus (Dentsply Sirona, Konstanz, Germany). To facilitate evaluation of multiple parameters, each main group was further subdivided into three equal subgroups (n = 8 per subgroup). These subgroups were specifically designated for separate assessment of dentinal tubule penetration, void formation, and push-out bond strength.

Obturation procedure

All root canals were obturated using the single-cone technique with F3 gutta-percha cones (Dentsply Sirona, Ballaigues, Switzerland) corresponding to the final instrumentation size. The sealer was introduced into the canal using a manufacturer-provided delivery syringe tip (for premixed bioceramic sealers) or a lentulo spiral (AH Plus). The master gutta-percha cone was lightly coated with a sealer and gently seated at the working length. Excess coronal gutta-percha was removed using a heated instrument and was vertically condensed. For all premixed injectable sealers, the manufacturer-provided syringe tip was inserted into the canal up to 2 mm short of the established working length, using the coronal reference point as the measurement guide. The sealer was delivered while slowly withdrawing the syringe tip in a continuous motion, ensuring uniform coating of the canal walls and minimizing air entrapment. This standardized intracanal placement depth was maintained for all specimens to ensure comparable sealer distribution across groups. Post-obturation radiographs were taken to confirm proper adaptation and the absence of significant voids. All specimens were then stored in an incubator at 37 °C and 100% relative humidity for two weeks to ensure complete setting of the sealers.

Evaluation of dentinal tubule penetration

**Figure 1:**
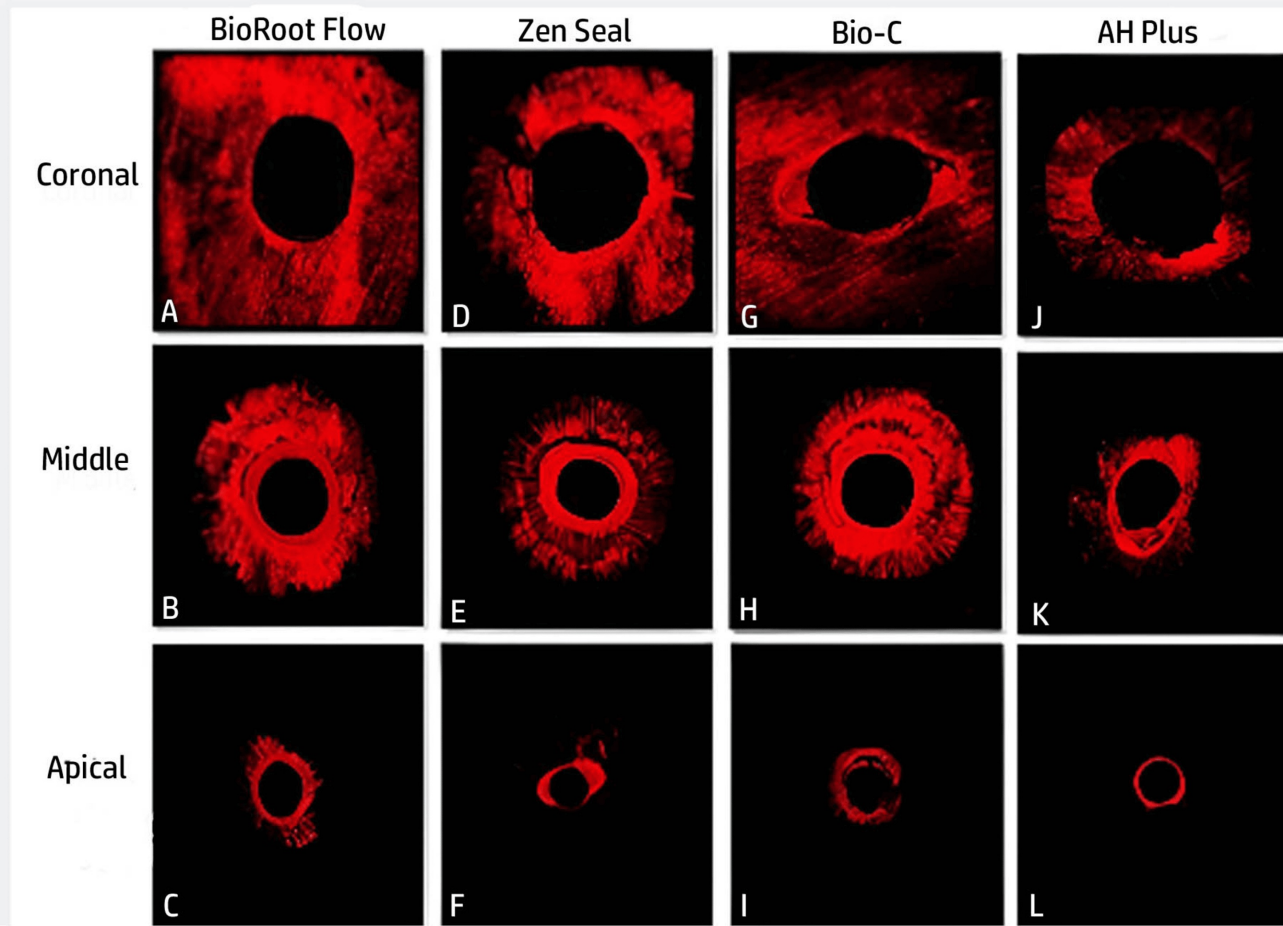
Representative confocal laser-scanning microscopy images (original magnification 10×) demonstrating the penetration depth of root canal sealers labeled with 0.1% Rhodamine B into dentinal tubules in the coronal, middle, and apical root thirds. Red fluorescence highlights sealer penetration from the canal wall. Panels A–C: BioRoot Flow; D–F: ZenSeal; G–I: Bio-C Sealer; J–L: AH Plus. Original images of samples from the study.

For this parameter, the assigned sealers were premixed with 0.1% Rhodamine B fluorescent dye (Sigma-Aldrich, St. Louis, MO, USA) prior to obturation. After the storage period, three 1-mm-thick transverse sections were prepared from the coronal, middle, and apical thirds of each root using a low-speed diamond saw under water cooling. The sections were embedded in epoxy resin, polished with progressively finer silicon carbide papers, ultrasonically cleaned, and mounted on glass slides. Examination was performed using a confocal laser-scanning microscope (Nikon, Melville, NY, USA) at 10× magnification with an excitation wavelength of 543 nm. The depth of fluorescent sealer penetration into the dentinal tubules was measured in micrometers (µm) from the canal wall in four quadrants per section (Figure 1).

Evaluation of void formation

Following the two-week storage period, reference marks were made at 4, 8, and 12 mm from the apex. The roots were transversely sectioned into 3-mm-thick slices representing the apical, middle, and coronal thirds. The sections were observed under a stereomicroscope (Olympus, Tokyo, Japan) at 40× magnification. Digital images were captured and analyzed using ImageJ software (National Institutes of Health, Bethesda, MD, USA). The total area of voids (within the sealer, at the sealer-dentin interface, and at the sealer-gutta-percha interface) was quantified in square millimeters (mm²) using standardized image analysis protocols.

Evaluation of push-out bond strength

Three 1-mm-thick slices were obtained from the coronal, middle, and apical thirds of each specimen. Each slice was positioned on a custom-fabricated jig with the apical side facing downward. Push-out testing was performed using a universal testing machine (Instron E3000; Norwood, MA, USA) at a constant crosshead speed of 1 mm/min. Stainless steel plungers of appropriate diameter (slightly smaller than the canal diameter) were used to apply compressive force until bond failure, which was identified by a sudden drop in the load-displacement curve. The maximum load at failure was recorded in Newtons (N). The bond strength was calculated in megapascals (MPa).

Statistical analysis

Data were analyzed using IBM SPSS Statistics for Windows, Version 23.0 (released 2015, IBM Corp., Armonk, NY). The Shapiro-Wilk test confirmed the normal distribution of data for the measured outcomes: tubule penetration (in µm), void size (in mm²), and push-out bond strength (in MPa). Intergroup and intragroup comparisons were performed using one-way ANOVA, followed by post-hoc tests for significant results. Statistical significance was set at P < 0.05.

## Results

Analysis of dentinal tubule penetration revealed statistically significant differences between the sealer groups and across root sections (p < 0.001 for all comparisons). Between groups, BioRoot Flow demonstrated the highest penetration depth at all levels (coronal: 1199.43 ± 104.75 µm; middle: 990.95 ± 116.69 µm; apical: 709.65 ± 121.99 µm), followed by Bio-C, ZenSeal, and finally AH Plus, which consistently showed the lowest values. Within each sealer group, a significant gradient was observed, with progressively decreasing penetration from the coronal to apical section (p < 0.001). This pattern indicated that all materials were subjected to anatomical and technical constraints in the narrower apical region. The inference is that the bioceramic sealer BioRoot Flow possesses superior penetrative ability into dentinal tubules compared to the other materials tested, including epoxy resin-based AH Plus (Table 1). Post-hoc pairwise comparisons confirmed that BioRoot Flow had significantly greater penetration than all other sealers in every section. Bio-C also surpassed ZenSeal and AH Plus, whereas ZenSeal outperformed AH Plus in all regions. Within each group, penetration in the coronal third was significantly higher than that in the middle, which, in turn, was higher than that in the apical third (p < 0.001 for all comparisons), establishing a clear material performance hierarchy and anatomical gradient.

**Table 1:** Comparison of dentinal tubule penetration depth (µm) of four root canal sealers (BioRoot Flow, ZenSeal, Bio-C, and AH Plus) at the coronal, middle, and apical root sections. Values are expressed as mean ± standard deviation (SD), between-group and within-group comparisons at each root level were performed using one-way analysis of variance (ANOVA), F indicates the F-statistic obtained from ANOVA, *p < 0.05 was considered statistically significant.

Section	BioRoot Flow	ZenSeal	Bio-C	AH Plus	F (between group)	p-value
Coronal	1199.43 ± 104.75	974.75 ± 59.00	1084.37 ± 74.80	814.43 ± 66.67	105.35	0.001*
Middle	990.95 ± 116.69	851.66 ± 80.68	877.22 ± 93.08	632.83 ± 95.92	56.42	0.001*
Apical	709.65 ± 121.99	658.19 ± 78.94	690.28 ± 73.24	428.91 ± 96.50	45.54	0.001*
F (within group)	110.2	113.03	142.59	116.66	-	
p-value	0.001*	0.001*	0.001*	0.001*		

The analysis of the void area revealed highly significant differences between the sealer groups and across root sections (p < 0.001). Between the groups, AH Plus demonstrated substantially larger voids at all levels (such as coronal: 0.0346 ± 0.0045 mm²), exceeding the bioceramic sealers by an order of magnitude. Among the bioceramic materials, BioRoot Flow and Bio-C consistently exhibited the smallest void areas. Within each group, a significant decrease in void size was observed from the coronal to apical section (p < 0.001). This pattern suggests that void formation is influenced by both the material properties and root canal anatomy. The inference is that the epoxy resin-based AH Plus sealer is prone to significantly greater void formation than contemporary bioceramic alternatives (Table 2). Post-hoc tests revealed that AH Plus exhibited a significantly larger void area than all bioceramic sealers in every root section (p < 0.001). Among the bioceramics, BioRoot Flow and Bio-C generally showed statistically smaller voids than ZenSeal, particularly in the middle and apical thirds (p < 0.05). Within each material, the void area in the coronal section was significantly larger than that in the apical section (p < 0.001), highlighting a consistent pattern of void reduction from the coronal to the apical region across all groups.

**Table 2:** Comparison of the area of voids (mm²) produced by BioRoot Flow, ZenSeal, Bio-C, and AH Plus at the coronal, middle, and apical root sections. Values are presented as mean ± standard deviation (SD). One-way ANOVA was used for between-group and within-group comparisons at each root section, F represents the ANOVA F-value, *p < 0.05 was considered statistically significant.

Section	BioRoot Flow	ZenSeal	Bio-C	AH Plus	F (between group)	p-value
Coronal	0.0056 ± 0.0007	0.0083 ± 0.0008	0.0060 ± 0.0008	0.0346 ± 0.0045	858.63	0.001*
Middle	0.0030 ± 0.0019	0.0043 ± 0.0027	0.0035 ± 0.0022	0.0230 ± 0.0051	217.09	0.001*
Apical	0.0011 ± 0.0005	0.0042 ± 0.0018	0.0023 ± 0.0010	0.0120 ± 0.0029	178.85	0.001*
F (within group)	84.47	35.26	39.59	168.21	-	
p-value	0.001*	0.001*	0.001*	0.001*		

Push-out bond strength analysis revealed statistically significant differences between the sealer groups and root canal sections (p<0.001). Between groups, the epoxy resin-based AH Plus demonstrated the highest bond strength at all levels (coronal: 2.19 ± 0.22 MPa; apical: 1.36 ± 0.31 MPa). Among the bioceramic sealers, BioRoot Flow consistently exhibited the highest values, followed by those of ZenSeal and Bio-C. A highly significant gradient was observed within each group, with bond strength decreasing progressively from the coronal third to the apical third (p<0.001), reflecting the anatomical challenge of achieving adhesion in the constricted apical region. While AH Plus provides superior immediate adhesive strength, potentially from its micromechanical bonding, bioceramic sealers, particularly BioRoot Flow, still offer substantial adhesion. This performance must be balanced against the superior penetration and lower void formation. The apical region remains a critical zone, where all materials show significantly reduced retention, highlighting a universal limitation in achieving optimal bond strength in the most challenging part of the root canal system (Table 3). Post-hoc analysis indicated that AH Plus possessed a significantly higher bond strength than all bioceramic sealers at each root level (p<0.001). Among bioceramics, BioRoot Flow demonstrated significantly greater strength than ZenSeal and Bio-C (p<0.05). For all materials, the bond strength in the coronal third was significantly superior to that in the middle, which was, in turn, stronger than that in the apical third (p<0.001), confirming a universal and significant decrease in adhesion from the coronal to apical direction.

**Table 3:** Comparison of push-out bond strength (MPa) of BioRoot Flow, ZenSeal, Bio-C, and AH Plus at coronal, middle, and apical root sections. Data are expressed as mean ± standard deviation (SD). Between-group and within-group differences were analyzed using one-way ANOVA. F denotes the F-statistic. Statistical significance was set at *p < 0.05.

Section	BioRoot Flow	ZenSeal	Bio-C	AH Plus	F (between group)	p-value
Coronal	1.92 ± 0.08	1.73 ± 0.19	1.72 ± 0.19	2.19 ± 0.22	36.6	0.001*
Middle	1.58 ± 0.08	1.36 ± 0.27	1.32 ± 0.26	1.82 ± 0.33	19.91	0.001*
Apical	1.06 ± 0.05	0.89 ± 0.10	0.87 ± 0.15	1.36 ± 0.31	37.61	0.001*
F (within group)	882.82	107.23	103.17	49.13	-	
p-value	0.001*	0.001*	0.001*	0.001*		

## Discussion

The findings of this study highlight the distinct performance profiles of the tested premixed injectable bioceramic sealers and the conventional epoxy resin-based sealer AH Plus. BioRoot Flow exhibited superior dentinal tubule penetration, minimal void formation, and relatively high push-out bond strength, suggesting that it is a promising alternative to AH Plus in clinical scenarios that prioritize adaptation and sealing efficacy. These differences can be attributed to inherent material properties: bioceramic sealers are hydrophilic, leveraging moisture for hydraulic setting and hydroxyapatite formation, which enhances flowability and interfacial integration with dentin [4]. By contrast, AH Plus relies on hydrophobic resin chemistry for micromechanical interlocking, offering robust adhesion, but limited performance in moist environments or with procedural inconsistencies. A previous study has reported that calcium-based sealers demonstrated the greatest dentinal tubular penetration, whereas AH Plus demonstrated the highest push-out bond strength [9]. 

Although endodontic sealers are not intended to function as luting agents and gutta-percha provides the primary core, push-out bond strength is clinically relevant as it reflects the sealer’s ability to maintain interfacial integrity and resist dislodgement from canal walls under functional and procedural stresses. This parameter represents the combined effects of sealer-dentin adhesion, mechanical interlocking, and frictional resistance within the obturation complex, which are essential for preserving the continuity of the apical and coronal seal. Therefore, push-out bond strength serves as a standardized surrogate measure of obturation stability rather than cementation capability.

Dentinal tubule penetration was significantly greater for BioRoot Flow across all root thirds, followed by Bio-C and ZenSeal, with AH Plus showing the lowest penetration. This gradient, decreasing apically, aligns with anatomical constraints such as narrower tubules and reduced irrigant access in the apical region, which impedes sealer flow. The superior penetration of bioceramics stems from their fine particle size, low viscosity, and hydrophilicity, which enable deeper infiltration into tubules and block microbial pathways more effectively. Supporting this, a study evaluating Bio-C Sealer versus AH Plus reported enhanced tubule penetration for bioceramic due to its premixed formulation and bioactive interaction with dentin, resulting in a mineral infiltration zone that improves sealing [10]. A systematic review and meta-analysis included 54 studies and concluded that both bioceramics and AH Plus demonstrated similar penetration ability in dentinal tubules and antimicrobial efficacy in coronal, middle, and apical thirds [11]. Another investigation confirmed that premixed bioceramics, such as BIO-C ION+, achieved maximum penetration compared to AH Plus, emphasizing the role of flowability in overcoming apical challenges [12]. These findings underscore that, while AH Plus's hydrophobicity limits penetration in moisture-rich canals, bioceramics exploit this environment for superior tubule occlusion, potentially reducing reinfection risks.

Void formation was markedly higher in AH Plus, with bioceramics, particularly BioRoot Flow and Bio-C, showing significantly smaller voids. The decreasing void size from coronal to apical may reflect better sealer compaction in narrower segments; however, overall, premixed injectable delivery minimizes air entrapment and inconsistencies from manual mixing, a common issue with AH Plus's two-paste system. Voids compromise seal integrity, permitting fluid leakage and bacterial ingress; thus, reduced voids in bioceramics enhance hermetic obturation. Corroborating evidence from a micro-CT study on BioRoot RCS, MTA-Fillapex, and Bio-C indicated lower void volumes for bioceramics versus resin-based sealers, which is linked to their consistent viscosity and single-cone compatibility [13]. Another comparison using continuous wave condensation with bioceramic sealers reported superior obturation quality and fewer voids than single-cone techniques with conventional sealers, highlighting technique-material synergy [14]. These results suggest that AH Plus technique sensitivity, including bubble incorporation during mixing, exacerbates voids, whereas premixed bioceramics offer procedural reliability.

The push-out bond strength was the highest for AH Plus, followed by BioRoot Flow, with a universal decline apically due to reduced surface area and dentin quality. AH Plus epoxy resin enables strong covalent bonding and tag formation into tubules, providing resistance to dislodgement under functional loads. Bioceramics, while chemically bonding via apatite, may yield a lower initial strength but improve over time through bioactivity. This is supported by a study in which AH Plus demonstrated maximum bond strength over BIO-C ION+ and NanoSeal-S despite the latter's better penetration, indicating the superiority of resin-based adhesion in shear tests [12]. Another evaluation found that AH Plus was superior to bioceramics, such as BioRoot, which exhibited substantial adhesion and balanced penetration benefits [15]. A systematic review by Silva et al. [16] concluded that epoxy resin-based sealers demonstrate greater push-out strength than premixed ready-to-use calcium silicate-based root canal sealers. However, a previous study has demonstrated that phosphate-rich media, such as phosphate-buffered saline, significantly enhanced the bioactivity of bioceramic sealers, resulting in increased apatite formation [17]. 

Clinically, the advantages of bioceramics in penetration and void reduction could promote better periapical healing, especially in moist or complex canals, although AH Plus's bond strength suits high-load scenarios. Limitations include the in vitro design, lack of dynamic oral conditions, such as thermal cycling or microbial challenges, and the use of the single-cone technique, which may not generalize to warm methods. Future studies should assess the long-term outcomes and biocompatibility in vivo.

## Conclusions

This in vitro study demonstrated that premixed injectable bioceramic sealers, particularly BioRoot Flow, significantly outperformed the conventional epoxy resin-based sealer AH Plus in dentinal tubule penetration and void reduction across all root thirds while maintaining clinically acceptable push-out bond strength. These superior sealing characteristics, such as deeper tubule occlusion, minimal voids, and enhanced adaptation, are attributed to the hydrophilic nature, fine particle size, and bioactive properties of the bioceramic materials. Although AH Plus exhibited the highest immediate bond strength, the overall performance profile of BioRoot Flow suggests that it is a promising alternative for achieving a more reliable, hermetic obturation. These findings support the increasing clinical use of premixed bioceramic sealers in modern endodontics.
